# Explanatory models for the cause of Fragile X Syndrome in rural Cameroon

**DOI:** 10.1002/jgc4.1440

**Published:** 2021-06-17

**Authors:** Karen Kengne Kamga, Jantina De Vries, Séraphin Nguefack, Nchangwi Syntia Munung, Ambroise Wonkam

**Affiliations:** ^1^ Division of Human Genetics Department of Pathology University of Cape Town Cape Town South Africa; ^2^ Department of Medicine Faculty of Health Sciences University of Cape Town Cape Town South Africa; ^3^ Department of Pediatrics Faculty of Medicine and Biomedical Sciences University of Yaoundé 1 Yaoundé Cameroon; ^4^ Institute of Infectious Disease and Molecular Medicine (IDM) Faculty of Health Sciences University of Cape Town Cape Town South Africa

**Keywords:** Cameroon, causal beliefs, community, explanatory model, Fragile X Syndrome, genetic counseling

## Abstract

Among the myriad causes of intellectual disability (ID), Fragile X Syndrome (FXS) is the leading genetic cause. Yet, little is known of how people affected by this condition make sense of it. The present study aimed to investigate the explanatory models for the causes of FXS in an extended family mainly affected by this condition and members of the village from which they originated in Cameroon. Using an ethnographic approach, 92 participants were interviewed (59 females and 33 males) through 10 focus group discussions and 23 in‐depth interviews between April 2018 and February 2020. Data analysis revealed four explanatory models regarding the etiologies of FXS in the community. Firstly, the curse model described a curse from the chief because of the belief that his wives did not mourn his intellectually disabled servant. Secondly, the spiritual model relates FXS to a punishment from God. Thirdly, the socioeconomic model attributes FXS to events in the prenatal and perinatal periods. Finally, the genetic model describes the pattern of inheritance of the disease in the family. This paper helps to understand the explanatory disease models that exist for FXS in rural Cameroon and could inform genetic counseling practices, community genetic education, and policymakers when drafting protocols for public engagement activities.

**What is known on this topic**

In the quest for a diagnosis for Fragile X Syndrome (FXS), it is not unusual for parents and caregivers to develop non‐biomedical explanations for the disability of their children. Yet, little is known about what type of explanatory models African populations have with regards to FXS.

**What does this paper adds to the topic**

This paper clearly establishes how an African community discusses the causal beliefs for Fragile X Syndrome (FXS) in an affected family from a rural community in Cameroon. Through ethnographic methods, it describes the various explanatory models of FXS that are held by family and community members.

## 1 INTRODUCTION

Fragile X Syndrome (FXS), also known as the most common inherited cause of intellectual disability (ID), is a neurogenic condition that affects twice as many males as females. It is characterized by mild to moderate ID, autistic features, and dysmorphic signs such as large ears and testis (Coffee et al., 2009; Hunter et al., 2014; Parker et al., 2010). Its pathophysiology is related to an expansion of the Cytosine–Guanine–Guanine (CGG) repeat at the 5' non translated region of the Fragile X Mental Retardation gene 1 *(FMR1*) found on the X chromosome (Jin & Warren, 2000; Santoro et al., 2012). This CGG repeat expansion is transmitted through a dominant X‐linked mode of inheritance. Hence, affected males will transmit the gene to all their daughters, while females have a fifty percent chance to pass the trait on to their offspring (Peprah, 2012; Santoro et al., 2012). In Cameroon, in the pediatric neurology unit at the Yaoundé gyneco‐obstetric and pediatric hospital, Nguefack et al. (2013) showed that among the children who presented with developmental delays, 1.3% were due to a genetic cause. Among these genetic causes, FXS was the leading single‐gene cause of developmental delay identified (Nguefack et al., 2013).

Cameroon is a lower‐middle‐income country with an estimated population of over 25 million as of 2018. Poverty in Cameroon has increased by 12% between 2007 and 2014, and close to 60% of the people living in poverty concentrate in rural areas. Without universal health coverage, patients depend on their family members for financial support, and they also visit traditional healers in ill health (The World Bank, 2019; Wonkam et al., 2014). Like many other developing countries, Cameroon is experiencing the challenges of managing patients with chronic non‐communicable diseases, some of which have a genetic origin. Wonkam et al. argue that despite this shift toward managing patients with genetic diseases, physicians and medical students have little knowledge of genetic diseases and genetic testing and suggested that their knowledge could increase with the presence of a genetic service in Cameroon (Wonkam et al., 2006; Wonkam, Tekendo, et al., 2011). In this light, in 2011, two male siblings received a positive molecular diagnosis of FXS at the Yaoundé gyneco‐obstetric and pediatric hospital, Cameroon. Informal data collected at the first consultation from the mother of the two boys indicated that several members of her family related FXS to a curse from the founder of their family. This resulted in a cascade testing that confirmed the pattern of X‐linked dominant transmission in the family (Kengne, Nguefack, et al., 2020).

The causal beliefs of intellectual disability by affected individuals and their families could be ways communities interpret and organize illness in their society (Helman, 2007; Sobo & Loustaunau, 2010). The so‐called explanatory models (EM) are derived from the Common Sense Model (CSM) described by Diefenbach and Leventhal (1996). The CSM model describes how patients experience chronic, stressful events in five ways, which are as follows: having a name for the condition; knowing how long it will last; identifying the physical, psychological, and social consequences of having the condition as well as developing a curative model for the infirmity (Diefenbach & Leventhal, 1996). Individuals facing a chronic medically unexplained symptom tend to rely on non‐biomedical explanations to provide meaning to their condition (Frostholm et al., 2007; Sumathipala et al., 2008). In the quest for a diagnosis for FXS, it is not unusual for parents and caregivers to develop non‐biomedical explanations for the disability of their children due to the long lag time between the identification of the disability and the final diagnosis. In a summary of the available evidence, Kengne et al. (2020) describe that these non‐biomedical explanations usually appear to be developed when caregivers are grieving and describe situations where parents link FXS to divine causes (Kengne, De Vries, et al., 2020; Michie & Skinner, 2010). In their review, Bhikha et al. (2012) found that there is limited research in developing countries, most notably in Africa, concerning explanatory models of illnesses (Bhikha et al., 2012), and to the best of our knowledge, no African study exploring the explanatory model for FXS has been conducted before. In an attempt to fill this gap, we use qualitative research methods to explore the perception of the causes of FXS in a rural community of Cameroon, previously described (Kengne, Nguefack, et al., 2020). The village where this work was conducted has an unusually high incidence of FXS because of a founder effect. Our work aimed to investigate the range of explanatory disease models about FXS specifically, and ID, more broadly.

## 2 METHODOLOGY

### 2.1 Setting

This study was conducted in the community of the patient (P0) who received a genetic diagnosis of FXS for her sons. P0 is a 48‐year‐old mother of three children, two of whom were diagnosed with FXS, the first two boys described in the introduction, and as previously reported (Kengne, Nguefack, et al., 2020). She originated from a small rural village in the Western region of rural Cameroon, from a family that has an unusually high incidence of FXS. This high incidence is due to a so‐called founder effect (Kengne, Nguefack, et al., 2020). People in this village practice agriculture for a living, and more than 50% of the villagers live in poverty. The village is situated about 10km from the main hospital in the region. However, it has a community health clinic that provides primary health care. Support services for children with developmental disabilities are rare in the country. The few services that are present are in the two capital cities: Douala and Yaoundé, which are situated more than 400km from the village. Hence, adequate care is not available to most villagers affected by FXS. In this setting, the spoken languages are French, English, Pidgin, and Ngombalé.

### 2.2 Study design

In 2011, P0 sought a clinical diagnosis for the condition of two of her children from two of the authors of this manuscript (SN and AW). After receiving the diagnosis of FXS, she shared with her healthcare providers what people in the village, where she originates, believe about FXS. Many years later, and following discussions with P0, we conceived this research project to better understand explanatory disease models used for FXS in the community and the family. It so happens that P0’s family is the founding family of the village that still holds the Chieftaincy. As such, they are a politically powerful and well‐known family in the village. This project was part of a larger project which seeks to investigate and understand the effect of receiving individual genetic results in Africa, named IFGENERA, standing for Individual findings in Genetics Research in Africa (Wonkam & de Vries, 2020). Given the enthusiastic, collaborative will of P0 and her family, we decided to use an ethnographic approach (Reeves et al., 2008) to carefully build relationships and understand the dynamics around FXS in the village. P0 introduced us to her extended family and to key opinion makers in the community from which she originates. We interacted with this community for two years. During that time, we sought permission to conduct research in the extended family as well as the village, engaged with stakeholders over multiple events and meetings, developed and piloted our research instruments, and conducted our research.

### 2.3 Study participants and sample selection

Snowball sampling was used to recruit family and community members (Noy, 2008). This sampling strategy was to ensure that participants were knowledgeable informants and reflected a range of characteristics of individuals potentially impacted by FXS in the family and community of P0. Following our introduction into the family, the family leader—who is the current Chief of the village—as well as other senior members of the family were contacted. When the agreement was obtained from the family to participate in the research, they invited us to present the research project at their annual family reunion in 2018 and 2019. During these meetings, family members were invited to participate in the study voluntarily. The details of interested individuals were collected and were later telephonically called to schedule the interviews at a date and place of their preference.

Once the participation of the extended family was secured, we proceeded by seeking community permission for participation. The Chief and elders had already consented, but we also required permission from district authorities as well as key opinion leaders in the village. Following a series of community engagement events to secure permission to recruit in the village, community members were invited to participate in focus group discussions through public announcements and personal invitations.

Participants were men and women, 18 years of age, and older. Participants with FXS were not recruited. Empirical data were collected until saturation, where further interviews did not yield new insights (Fusch & Ness, 2015). A total of 23 in‐depth interviews (IDIs) and 10 focus group discussions (FGDs) were conducted. The number of participants in FGD varied between 4 and 11 and were classified either as community members or relatives of P0.

### 2.4 Procedure for data collection

All interviews were conducted between August 2018 and February 2020. The IDIs and FGDs were conducted either in French or English, depending on the first language of the participant. However, during FGDs, some participants expressed themselves in the local language (Ngombalé), which was translated during transcription but recorded in the vernacular in our field notes as a ‘memo’. All the IDIs lasted between 27 and 60 min while the FGDs lasted between 45 and 90 min. All sessions were audio‐recorded, and topic guides were used to guide the discussions with participants to understand their perceptions of, among others, lived experiences with, the stigma associated with, and the effects of receiving a genetic diagnosis of FXS. At the end of every interview, a genetic information session was offed to participants where all their questions concerning FXS and its mode of inheritance were answered. Participants were given financial compensation for their time and transport. Participants were assured confidentiality, as much as possible, by referring to individuals interviewed in groups with identification numbers rather than their names. In cases where participants knew each other, they were asked if they are willing to share their information with members of the FGD; otherwise, they could participate in the in‐depth interview. All interviews were digitally recorded and transcribed verbatim. The topic guide is provided as Supporting information.

### 2.5 Analysis

Digital recordings from individual and group interviews were cleaned of identifying information, and transcriptions were translated from French into English as needed. Transcriptions were reviewed for accuracy then imported, with the researcher's memos and field notes, into NVivo 12 qualitative data management program (www.qsrinternational.com). Inductive coding was used to identify themes emerging from the data (Thomas, 2006). Thematic analysis was used (Braun & Clarke, 2012) to identify patterns of meaning across the dataset, both within‐case and between‐cases, as we read through the transcripts. The analysis involved the search for understanding of similarities and differences among participants concerning knowledge and views related to FXS. The first round of coding was done by KKK when approximately two‐thirds of the data was collected. The codes developed, and the memos were discussed with JDV and NSM to ensure that they accurately captured critical details from the transcripts. Insights from this early phase of data analysis guided the final stage of data collection and helped us ensure saturation was reached. After obtaining a complete dataset, KKK and JDV collectively developed the hierarchical coding scheme that was applied to the full dataset. KKK coded the full dataset, and through consensus, the study team identified the primary themes that were related to the explanatory models for the causes of FXS, as perceived by participants.

## 3 RESULTS

### 3.1 Description of the sample

A total of 92 participants agreed to be part of our study, of which 64% were females, and 36% were males. The median age of participants was 42 years (range: 18–81 year). Sixty‐three out of the 92 participants were community members, while 29 were family members. The majority of the participants had a secondary level of education, and more than 75% were unemployed (Table 1). Participants who said they were unemployed lived on agriculture or trading while employed participants were either teachers, religious leaders, or traditional leaders.

**TABLE 1:** Demographic characteristics of our population

Variable	Percentage (%)
Gender	
Male	36
Female	64
Level of education	
Primary	28
Secondary	59
University	13
Profession	
Employed	24
Unemployed	76

### 3.2 Name of FXS in the community

**FIGURE 1:**
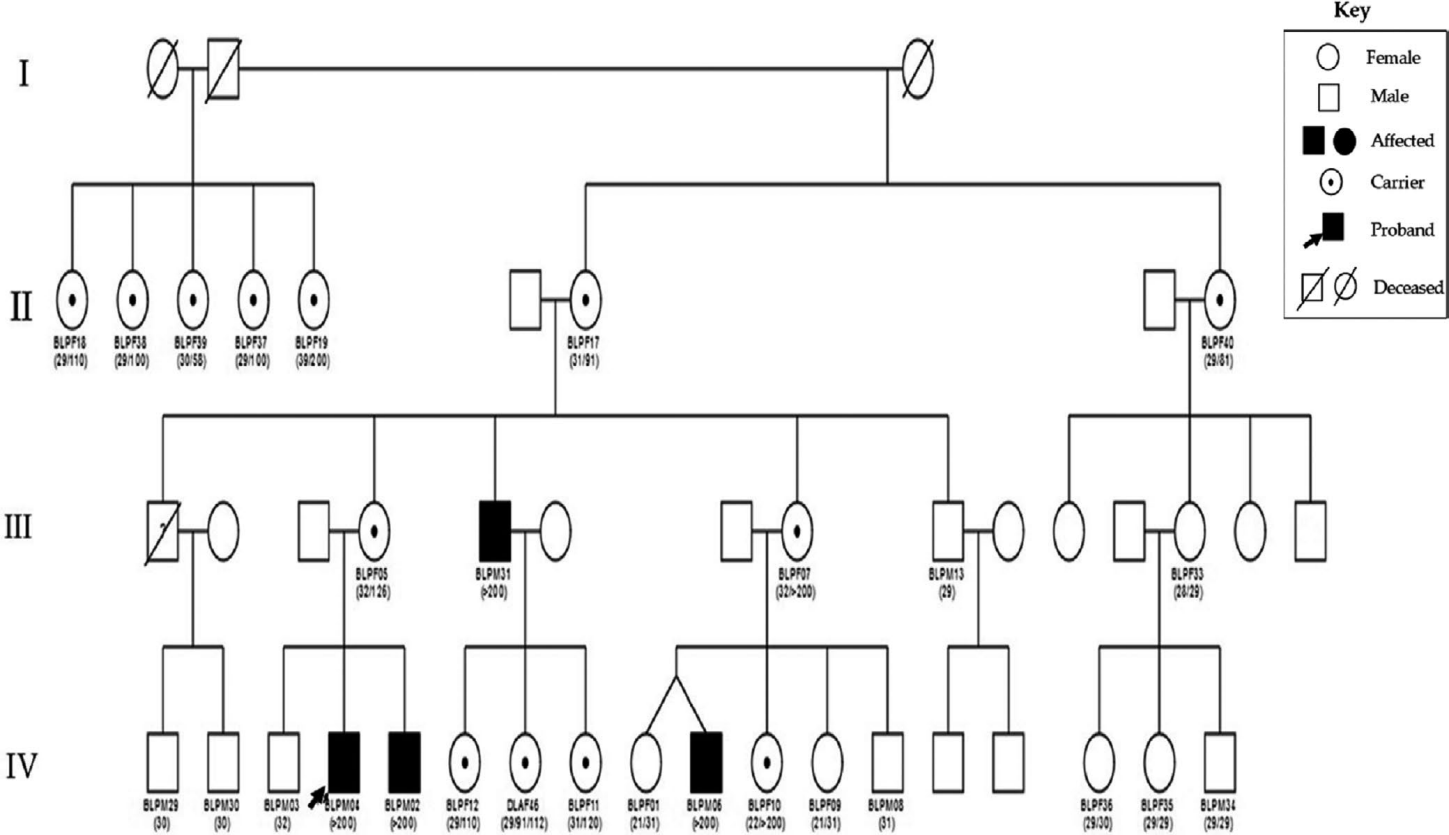
Pedigree of P0 extended family. The number in brackets below the symbols represent the number of CGG repeats in the FMR1 gene. This figure was retrieved from our previous publication with the title; Cascade testing for FXS in a rural setting in Cameroon sub‐Saharan Africa. (Kengne, Nguefack, et al., 2020)

Overall, community members appeared to have come to the view that the family of patient P0—the royal family in the village—was susceptible to having children with FXS (Figure 1). They distinguished between FXS and ID. For instance, when speaking of FXS, participants either used ‘alienate’ or ‘rheurheu’ to describe people with inheritable forms of ID like FXS. In contrast, other forms of ID were referred to as madness, follies, or ‘Peuh’. An alienate or a ‘rheurheu’ is described as behaving strangely, with fluctuating moods, but is able to live just like any other person. A female participant explained this in a FGD:

There is pure madness that we already know. However, when it is an alienate who was born like that, they still think a little. There are times when they go off their senses before regaining it again. I can say that this is the type who wants to isolate themselves. The family does not isolate him because they are alienates; they [alienates] do so on their own. (FGD, Community 1, Female)

This discussion was followed up with other community members who gave us the meaning of these different names. These explanations were recorded as memos.

In the local language, ‘rheurheu’ means a child who is an Alienate, while ‘Peuh’ means madness. Participants reported that their children are not mad but somewhat alienated. They preferred that we used ‘rheurheu’ in subsequent interviews. This is because ‘rheurheu’ is ‘better’ than ‘peuh’ because you can take care and support a ‘rheurheu’ until he/she even gets married while you cannot do anything for a mad man. (Field notes 15 October 2018)

### 3.3 Perception of the causes of FXS in the community

**FIGURE 2:**
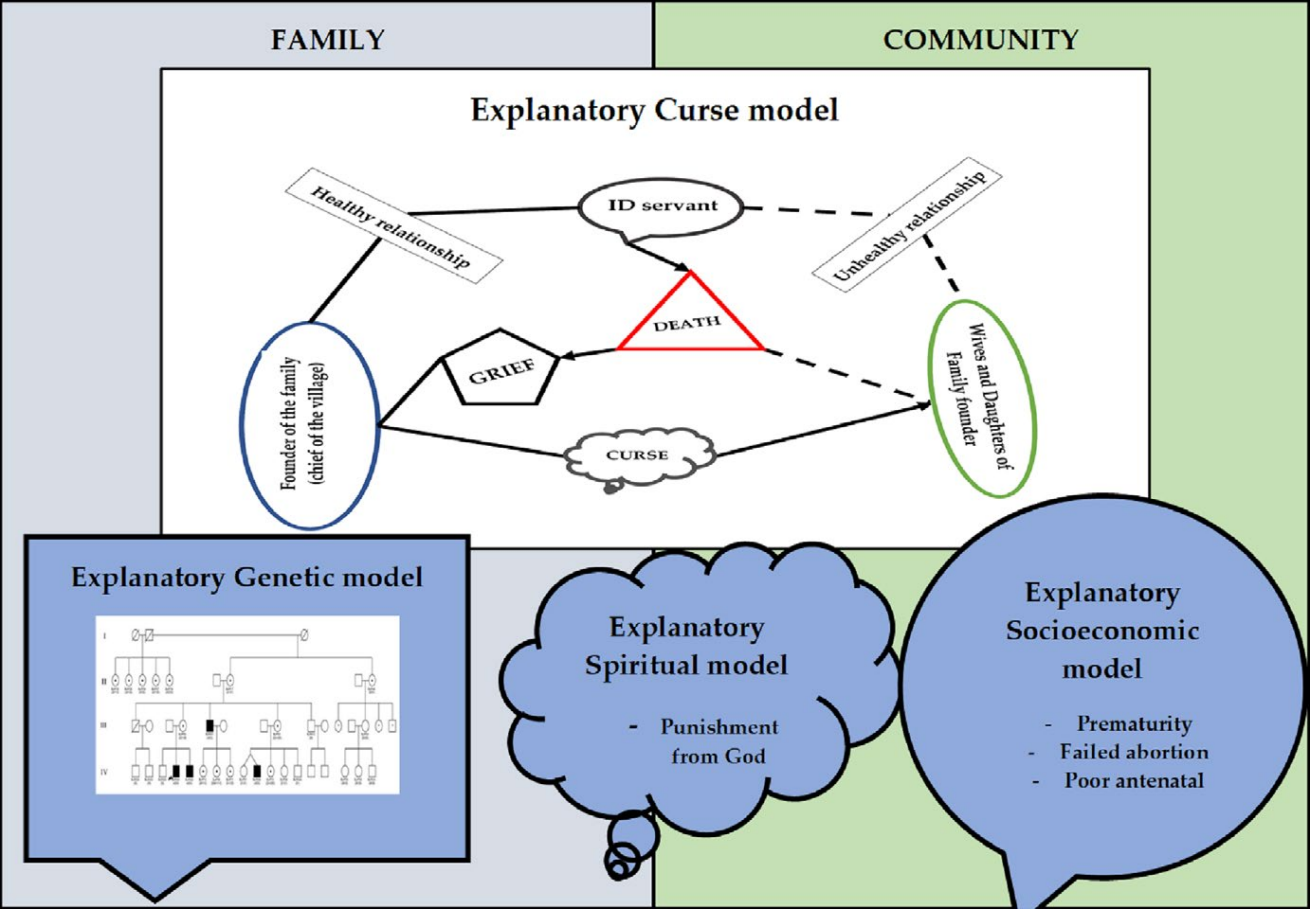
Map describing the different explanatory models used in P0’s family and community for the cause of FXS

In an attempt to understand why there is a high incidence of FXS in P0’s family, community members proffered four possible explanatory models: the curse model, the spiritual model, the socioeconomic model, and the genetic model (Figure 2).

#### 3.3.1 The curse model

More than 75% of the interviewees spoke about the curse as an explanatory model of FXS in P0’s extended family. This social explanation is based on the hypothesis that the founder of family F0 (chief of village) had cursed his daughters for not assisting him in the preparation of the funeral ceremony of one of his servants. An elder of the village suggested that the relationship between the chief and this servant was a positive one, where the servant assisted the chief in all his activities like trading, splitting firewood for the wives, and fetching water.

So, the chief bought a slave who was alienated but was physically strong. He wanted his alienate servant to help his wives by splitting the wood and help him with his shopping. He lived for a long time with this alienate servant (IDI, community, An elder of the village)

On the other hand, the physical appearance of the servant did not impose the respect of the women in the palace. Rather, they usually mocked him and did not want to see him.

look how you are, look how dirty you are. Can't you have a bath? It is stuff like that. That is to say, when someone comes to my house, for example, and sees an alienated child; they start to say things that go in the direction of mocking the child’ (FGD, community 4, Female)

Considering the caring relationship that existed between the chief and his servant, the chief was furious when his wives and daughters did not assist in the funeral of the disabled servant as he had expected them to be. Instead, they went to the farms with their mothers. The village narrative related to us is that the chief then said that his daughters were going to have alienated children, just like his servant. A female in family F0 recounted this story that is widely accepted by the community and the family members:

It is said that when the ‘rheurheu’ died, the chief’s wives went to their farms even after knowing that the ‘rheurheu’ was dead they did not tell my father. While the women were on their farms, my father found out that the alienate had died. When the women returned, he asked them why they had not informed him of the death of his servant and why they went to their farms, leaving the servant’s corpse unattended. After that, he said: “One day, you too will give birth to a ‘rheurheu’. It is from this moment that we, the children of [the chief], started giving birth to ‘rheurheu’. This latest version seems real because my father cursed his wives and daughters. (FGD, Family 4, female)

This story has gone through several modifications over the years, and this final construct seems more acceptable to the community. However, other interviewees think that the curse is related to the reincarnation of the alienated servant in family F0. A male interviewee reported:

When he died [the alienated servant], the family did not take care of him, and over time we said to ourselves that this is a phenomenon of reincarnation and that it may be the reason why we have mentally deformed children. (IDI, Family, male 4)

It is worth noting that traditional customs support this argument since males who die without having children are thought to reincarnate within the family. Hence, it is thought that the curse in the community is the reincarnation of the alienated servant of the chief. ‘It is often said that the curse of people who have lived without having a wife or children is very dangerous’. (IDI, Family, male 5).

There were also some community members who were not aware of this royal curse, but who also described a belief that people with FXS had a curse placed on them to block their chances of evolving in society.

I think we are in Africa and we are Bantu. Very often, in some families, there is what is called the ‘curse’. We often throw this to some children to block their intelligence and their charisma. So, we would not like them to succeed, so, in my opinion, this can be the cause of inherited ID in some families. (FGD, community 5, Male)

#### 3.3.2 Spiritual model

Close to 20% of interviewees mentioned the explanatory spiritual model for the cause of FXS in the family. This model was recounted by religious leaders, some community members, and a small fraction of the relatives of patient P0. The spiritual model described FXS in terms of punishment of God. Using quotations from the bible, participants believed that the origin of people with FXS was a declaration from the founder of the family. Two male participants, one in the FGD and the other through IDI, argued:

When I read the Old Testament, I see that God had punishment in the form of diseases, terrors etc., for all sins. So, I accept that. I believe in my faith. I believe that if someone does something that is wrong, the consequences can fall on him or his descendants. (IDI, community, male 2)

Whether it is a curse from the parents or from God, the Bible says He punishes the iniquities of the parents up to the fourth generation. Also, we can be part of this generation that God cursed. So, if we are part of a generation that God has cursed, then what God said about our parents will affect us from generation to generation. If God pronounced the mental retardation curse, well, there are many things, be it poverty, illnesses, and so on, God can do that, and it affects us. (FGD, community 1, Male)

Some members of family F0 also shared this view and argued that this punishment is a consequence of the unfair treatment bestowed on the disabled servant. Hence, in order to prepare future generations of this situation, they were told not to mock intellectually disabled children. If they did, they would receive this punishment from God. Two members of the family elaborated:

It may be the work of God; it is bad luck on that side. However, as expressed in the bible, the gospel says, God punishes from the first or the second generation. We think it can be our punishment. (IDI, Family male 2)

However, we were already prepared for this because we knew that my father's sisters gave birth to alienate children. As their mother already knew what had happened, she always told me that I should never make fun of such a child because we do not know what God has in store for us. (IDI, Family, female 9)

So while the spiritual model significantly overlaps with the curse model, what is different is that in the spiritual model, participants see the curse as emanating from God.

#### 3.3.3 Socioeconomic model

The explanatory socioeconomic model for the causes of FXS was supported by about 20% of the interviewees as a possible cause for FXS, and all of them were community members. Members of family F0 did not attribute FXS to socioeconomic factors. The main socioeconomic models described were prematurity, inadequate health care, and the use of drugs during pregnancy. A mother in the community reported how the developmental delay of her child is associated with prematurity.

He was premature. He was born at six months, and he was 900 g in weight. So, he did not reach 1 kg. The child is now more than two years old; he does not sit down; he does not take anything by hand to eat. He has enough teeth, but he does not work well, he does not do the scrambling well like all children. So, he does not sit. (FGD, community 1, female)

Moreover, other community members reported inadequate care during the prenatal period as being the cause of FXS in the community. A male participant in the community elaborated on this and concluded that it was the lack of some injections that are given during the prenatal period and the poor follow‐up during the pregnancy that led to the occurrence of FXS:

We think that the cause must be the lack of certain injections during the Antenatal clinic and poor follow up. So, we can say at this point that one of the causes of these alienations may be the fact that the pregnant woman was not administered the required injections, which could positively influence the growth of the child's brain. (IDI, community, male 5)

Most interestingly, participants reported that FXS could be due to unlawful termination of pregnancy, notably if the woman used abortion‐inducing medication that failed to terminate the pregnancy.

It is said that there are some girls when they are pregnant, they take medication to abort. I do not know if these are traditional medicines or modern medicine. However, if the child does not go out and resists in the belly only coming out at nine months, the child grows until it starts to walk, then it loses the head. (FGD, community 4, Male)

#### 3.3.4 Genetic model

The explanatory genetic model is an emerging model that has not yet been integrated by family and community members. Only patient P0 and one of her cousins discussed this model. After the diagnosis of FXS in the patient P0's family, she was given genetic information related to FXS and the disability of her children.

Then he said it is a genetic problem because he asked for the family tree, and he now drew it and explained to me that my grandfather was this person that had many wives; the mother was this person. So, when I was explaining, he was drawing the family tree until he came out with the conclusion. He told me what was wrong with these children would be this thing (FXS), and that it comes from my mother's side, from my mother's paternal grandmother. (IDI, family, P0)

Following this diagnosis, patient P0 could talk to other family members who traced the origin of the condition to her great maternal grandmother, who was having some developmental problems just like her children. She then started doubting the curse model and developed a genetic model based on the information she received during the post counseling information session and her investigations. Her cousin recounts:

Recently, they got to know that it had nothing to do with their father killing a fool, that their father never killed a fool. That their grandmother, that is my mother's grandmother, looked somehow like an imbecile. That is where this trait is originating. (IDI, Family, P0’s cousin)

## 4 DISCUSSION

Our data suggest that in this Cameroonian rural community, people can identify genetic diseases and provide perceptions about the different causes that associate with FXS. Our findings are unique because this was the first study in Cameroon that explores the way cultures address children with inherited forms of ID, such as FXS. The names used to refer to children with ID have been reported in other African countries, like Ghana (Avoke, 2002; Opare‐Henaku & Utsey, 2017), but these studies refer to common names which translated to ‘being stupid or a fool’ for describing these children. However, Opare‐Henaku and Utsey (2017), in their analysis of the concepts used in the ‘Akan’ culture, found that there are different ways of addressing people with intellectual disability based on the severity and/or cause. Similarly, our study shows two names, ‘Rheurheu’ and ‘Peuh’, where ‘rheurheu’ is an inherited and milder form of ID which is more accepted by the community, while ‘peuh’ could be associated with severe forms of ID.

The community in describing FXS also described four explanatory models. We suggest that these models should not be regarded as a final explanatory model but rather as a map (Figure 2) of possible explanatory models for FXS in African communities, which represent the framework of an ongoing process in which the community uses to provide meaning for the causes of FXS. Some African scholars from Kenya and Nigeria indicate that ID can have a supernatural etiology (Bunning et al., 2017; Etieyibo & Omiegbe, 2016). These studies elaborate on jealousy and envy, as well as ancestral displeasure or curses, which were supernatural factors that led to IDs. It is also possible, with the increase of formal education in the community, that the genetic model could become dominant. Such trends were observed in Sickle cell disease (SCD), a genetic condition that is prevalent in Africa. Indeed, SCD cause was initially associated with a reincarnation model called ‘ogbanje’ in communities Nigeria, with malevolent ‘ogbanje’ that differs from others in being revenge‐driven, chronically ill and engaging in repeated cycles of birth, death, and reincarnation (Nzewi, 2001; Onwubalili, 1983). Poor knowledge of the disease has a potential impact on family dynamics. Indeed, in Nigeria, family ascribed the disharmony in their marriage to SCD in their children (Bamisaiye et al., 1974).

Furthermore, in rural Kenya, misperceptions regarding inheritance reinforced blaming patterns within families, and low initial recognition of SCD and its cause were associated with poor surveillance practices (Marsh et al., 2011). In contrast, more recent data from Nigeria, from which more than half of the parents had tertiary education, illustrates an improved knowledge of the heritable nature of SCD, and this had a beneficial effect on family dynamics (Brown et al., 2010). Similarly, in Cameroon higher education, occupational status and resources were associated with better knowledge of the heritable nature of SCD and this knowledge was a significant contributor to marriage stability and commitment to SCD‐affected children care (Wonkam et al., 2011).

Other models described in the community are the socioeconomic and genetic explanatory models. The socioeconomic models are related to events in the prenatal period. Nguefack et al. (2013) showed that there are several causes of developmental delay, with the main etiologies being related to perinatal and antenatal causes. The manifestations of FXS could mimic those of other perinatal causes of developmental delay, which results in the delay of its diagnosis (Christianson et al., 2002; Nguefack et al., 2013).

While the diagnosis of FXS for the heritable form of ID in the rural community of Cameroon has not yet changed the perception of people concerning ID in the village, it has given rise to a new explanatory model, the explanatory genetic model, that is starting to shed some doubts on the narrative of a curse in family F0. The reasons for the non‐generalization of the explanatory genetic model are yet to be explored in this family. Diefenbach and Leventhal (1996) argued that patients in the process of seeking the cause of intellectual disability could come across causes that are socially unacceptable and will prefer not to know (Diefenbach & Leventhal, 1996). This study supports the notion that people actively seek meaning for the experiences they face by holding onto a variety of explanations simultaneously. Due to the perceived uncertain cause of intellectual disability in the family, the community and family members utilized several non‐biomedical explanations. We can, therefore, suggest that these explanatory models for the cause of FXS are possibly a community construct used to cope with the high frequency of people with FXS in the rural community since they have high respect for traditional customs.

### 4.1 Practice implications

Our findings suggest that public health programs in Cameroon should not only aim at increasing knowledge and awareness about rare genetic conditions and its management. These programs should also elaborate on the relevance of genetic counseling, testing, and reproductive choice, specifically in the context of a heritable condition such as FXS. Strategies may include concerted efforts to educate families and community members about the genetic cause of Fragile X Syndrome, particularly in the present study setting. This could be achieved by involving and training local healthcare personnel on communicating genetic information through face‐to‐face explanation of Fragile X Syndrome, pedigrees analysis, and using information aides consisting of letters, brochures, and resource guides. These tools need to be prepared by the local physicians and nurses, who are more familiar with the different explanatory models within the community. With a broader knowledge base, community members and leaders might be persuaded to make use of available medical services for genetic counseling. Our findings also provide an insight into the challenges that could be addressed during the development and implementation of genetic counseling services; this may include the traditional knowledge of genetic diseases like FXS. Moreover, this study can also strengthen the current H3Africa ethics and community engagement guidelines and refine qualitative research strategies for our African context. We also show active engagement with a particularly sensitive community, suggest possible stigma due to a devastating and unknittable condition associated with intellectual disability, and fill a void in our understanding of African perspectives about ID and genetics.

### 4.2 Study limitations

Our study had a few limitations. The first limitation is related to recall bias because the explanatory model for FXS is mainly based on the participants’ ability to recall the different events that had happened. Secondly, our sample was mainly family relatives and literate community members. It could have been more comprehensive with the inclusion of the views of traditional healers of the village because they are the first people that community members will go to in the quest for a solution to their health problems. Thirdly, this study's ethnographic approach may constitute a limitation because participants could have presented exemplary behavior or told the researcher what they wanted to hear. The study results are only applicable in this setting since ethnographic research findings are particular to the research context in which a study was conducted and cannot be generalized.

### 4.3 Research recommendations

Future research should engage on how communities address the ethical and social implications of FXS and its associated health condition such as premature ovarian failure in carrier female, and Fragile X associated tremor ataxia syndrome (FXTAS) and reproductive health decisions, including the option of prenatal diagnosis which is now possible in Cameroon (Wonkam, Tekendo, et al., 2011). Moreover, whether the perception of explanatory models influence the commitment to the care of FXS‐affected individuals, as well as the coping mechanisms to its associated burden in families, will also need to be investigated. Besides, the association of the explanatory models and possible gender stigma associated with families whose female members are a potential carrier of FXS will need to be investigated.

## 5 CONCLUSION

This rural Cameroonian community has multiple ways of explaining the causes of FXS. The different explanatory models for the causes of FXS are a framework that will help the scientific community to understand the sociopolitical and cultural context of people living in the rural community. This knowledge is also an essential starting point for establishing a collaborative platform between health professionals, educators, and policymakers, who will significantly contribute to the development and evaluation of culturally and sensitive patient‐friendly interventions. Furthermore, cited perinatal events in this study draw our attention to the importance of developing programs for early detection of FXS through prenatal diagnosis, neonatal screening, and premarital diagnosis. As the field of genetic counseling focuses on diversity initiatives, including access to genetic testing and counseling services of underrepresented populations, studies such as the one explored in this paper will become increasingly crucial to informing these initiatives and improving genetic testing and counseling services to all.

## AUTHOR CONTRIBUTIONS

KKK involved in design, data collection, interpretation, and writing. NSM involved in interpretation and writing. SN, JDV, and AW involved in design, interpretation, and writing. KKK, JDV, and AW confirm that they had full access to all the data in the study and take responsibility for the integrity of the data and the accuracy of the data analysis. All of the authors gave final approval of this version to be published and agree to be accountable for all aspects of the work in ensuring that questions related to the accuracy or integrity of any part of the work are appropriately investigated and resolved.

## COMPLIANCE WITH ETHICAL STANDARDS

### CONFLICT OF INTEREST

The authors declare that they have no competing interests.

The funders had no role in study design, data collection, and analysis, decision to publish, or preparation of the manuscript.

### HUMAN STUDIES AND INFORMED CONSENT

The study was performed following the Declaration of Helsinki. Ethical approval for the study was obtained from the Institutional Committee for Health Research (no. 698/CIERSH/DM/2018) in Yaoundé, Cameroon, and the University of Cape Town's Faculty of Health Sciences’ Human Research Ethics Committee (HREC: 782/2017). Written informed consent was obtained from all participants who were all legal adults, including permission to publish photographs. Administrative authorizations were obtained from the local authorities (District Medical Officer and the village chief).

### DATA SHARING AND DATA ACCESSIBILITY

The data that support the findings of this study are available from the corresponding author upon reasonable request.

## Supporting information

Supplementary Material
