# Supplementary material for: Explanatory models for the cause of Fragile X Syndrome in rural Cameroon
Source: J Genet Couns. 2021 Jun 17;30(6):1727–36. doi: 10.1002/jgc4.1440 (PMC8642261; doi:10.1002/jgc4.1440)
Supplement: Supplementary file 1 — Supplementary Material [file JGC4-30-1727-s001.docx]

Topic guide

Return of a Fragile X Syndrome Genetic Result: Exploring the feedback of Individual genetic findings and their relation to traditional knowledge in a village in Cameroon

This document is a guide to the principal themes and issues to be covered. Questions can be modified and followed up in more detail as appropriate.

# Introduction (for the interviewer)

- Introduce yourself and thank them for receiving you or answering your appointment.

- Shear an outlook of the project with them and inform them that the interview will last for about 45 mins

- Remind them that participation is optional

- Establish ground rules

# Confidentiality

- Your names will not be tagged in the interview. You should feel free while sharing your opinions with us. If you feel like not answering any questions, you are free not to.

- We would prefer to record the interview as this helps us capture exactly what you said. Are you comfortable with that?

(Ask for verbal confirmation that they understand the purpose and confidentiality of the research and that they are happy to take part)

# Understanding of FXS in the village (use an opening question or an ice breaker to get the participant comfortable):

- Can you please tell me what you know about FXS in the village? (use the name that is common in the village)?

- Do you know if many people in the village have it? If yes

- Do you know how they contracted it?
- What could be the possible explanation for the causes of (use the name common in the village) in your community?

- Can you please give us a few narratives or stories around this phenomenon? (follow up)

- What about the story behind this belief? (use the name that is common in the village)?
- Do you have family members or relatives or friends with this condition (use the name common in the village)? (follow up)
- Can you please share with us your experience with them?
- How did they get this condition? (follow up)
- Are there other explanations for the high prevalence of this condition in the village?

# Diagnostic experience (for participants who received a FXS genetic test result)

- Tell us about your diagnosis experience, starting from when you first recognized any problems to when you got the diagnosis of fragile X syndrome for your child.

- What were some of the first symptoms that you noticed?

- What doctor gave you the diagnosis of FXS?

- What part of the whole process of receiving a diagnosis did you think went the smoothest?

- What part of the process was the most challenging?

- How did you feel when your child was given a diagnosis of fragile X syndrome?

- Tell us about any support you received when you first got the diagnosis?

- What role did your family play in the support you received?
- What role did your friends play in the support you received?
- Did you receive support from other types of organized groups?

- If you could change anything about the process of getting a diagnosis of fragile X syndrome, what would you change?

- Is there anything else you would like to tell us about your experience that might help us assist other families in the future?

# Diagnostic closure

- What made you go for a diagnostic test?

- What was it like when you first received the genetic results that your children had fragile x syndrome?

- What was the care given to the children before you knew about the medical test results? (follow up)

- How are they taken care of now?

- Did you inform other family members about genetic testing and results? Why do you think it was (or was not) important to let them know?

- (if we do a test and discover that there is fragile x syndrome (use the name that is common in the village) in your family, would you like us to tell you about the results? (Follow up) Why (why not)?

# Sigma and gender blame

- How have you been living with this condition? (use the name common in the village)

- How were peoples in the village react to people who have FXS? (use the name common in the village)

- Can you please give us some examples?

- How has this condition affected your relationship with relatives, other members of the community, or with the affected families?

# Closing notes

- Is there anything that we have did not address that you want to add?

- Thank you for your time
